# Ki-67 expression is superior to mitotic count and novel proliferation markers PHH3, MCM4 and mitosin as a prognostic factor in thick cutaneous melanoma

**DOI:** 10.1186/1471-2407-10-140

**Published:** 2010-04-14

**Authors:** Rita G Ladstein, Ingeborg M Bachmann, Oddbjørn Straume, Lars A Akslen

**Affiliations:** 1 The Gade Institute, Section for Pathology, University of Bergen, Bergen, Norway; 2 Department of Dermatology, Haukeland University, Hospital, Bergen, Norway; 3 Department of Pathology, Haukeland University Hospital, Bergen, Norway

## Abstract

**Background:**

Tumor cell proliferation is a predictor of survival in cutaneous melanoma. The aim of the present study was to evaluate the prognostic impact of mitotic count, Ki-67 expression and novel proliferation markers phosphohistone H3 (PHH3), minichromosome maintenance protein 4 (MCM4) and mitosin, and to compare the results with histopathological variables.

**Methods:**

202 consecutive cases of nodular cutaneous melanoma were initially included. Mitotic count (mitosis per mm^2^) was assessed on H&E sections, and Ki-67 expression was estimated by immunohistochemistry on standard sections. PHH3, MCM4 and mitosin were examined by staining of tissue microarrays (TMA) sections.

**Results:**

Increased mitotic count and elevated Ki-67 expression were strongly associated with increased tumor thickness, presence of ulceration and tumor necrosis. Furthermore, high mitotic count and elevated Ki-67 expression were also associated with Clark's level of invasion and presence of vascular invasion. High expression of PHH3 and MCM4 was correlated with high mitotic count, elevated Ki-67 expression and tumor ulceration, and increased PHH3 frequencies were associated with tumor thickness and presence of tumor necrosis. Univariate analyses showed a worse outcome in cases with elevated Ki-67 expression and high mitotic count, whereas PHH3, MCM4 and mitosin were not significant. Tumor cell proliferation by Ki-67 had significant prognostic impact by multivariate analysis.

**Conclusions:**

Ki-67 was a stronger and more robust prognostic indicator than mitotic count in this series of nodular melanoma. PHH3, MCM4 and mitosin did not predict patient survival.

## Background

Cutaneous melanoma is one of the most rapidly increasing malignancies among Caucasians [1,2], and improved understanding of its biological characteristics and prognostic factors is therefore important. Advanced stage disease is relatively resistant to conventional therapeutic approaches [3], and better insight in the molecular pathogenesis of melanoma development and progression could contribute to improved diagnostic tools and new strategies for targeted therapy. Since tumor cell proliferation is an increasingly important prognostic factor in many malignant tumors, its value in cutaneous melanoma has been examined in the present report by a comparison of several different markers.

The 2002 American Joint Committee on Cancer (AJCC) staging system for melanoma was based on a multicenter analysis of prognostic factors in more than 17,000 patients [4]. The pT classification included information on tumor ulceration, but not mitotic count [5]. In some recent studies, however, mitotic frequency is a powerful predictor of survival [6-11], and the inclusion of this marker in staging of primary melanomas has been included in the 2010 edition of the AJCC pTNM staging system.

The proliferation marker Ki-67 is expressed in all phases of the cell cycle [12], and elevated Ki-67 in tumor cells was associated with the most aggressive melanomas in our previous study [13]. A prognostic significance has also been shown in other studies [9], but results have not been consistent [12]. Although Ki-67 positivity is a marker of proliferative cells, it is uncertain how many of the cells expressing Ki-67 will actually undergo mitosis.

In 1997, the mitosis marker anti phosphohistone H3 was first introduced [14]. Phosphorylation of histone H3 (Ser 10) is shown to be closely associated with mitotic chromatin condensation in late G2 and M phase of the cell cycle [14,15], and histone H3 is not phosphorylated during apoptosis [16]. Subsequently, expression of PHH3 has been investigated in several cancers. Among brain tumors, PHH3 staining was primarily found to support grading by facilitating mitotic counting, but it also had a prognostic value [17,18]. In a series of lymph node negative breast cancer patients, PHH3 was the strongest prognostic variable [19]. In a small study of melanocytic lesions, PHH3 was a useful supplement in differentiating malignant melanoma from benign nevi, although it did not show any advantage compared to Ki-67 [20]. To the best of our knowledge, the prognostic value of PHH3 expression in malignant melanoma has not been previously evaluated.

In a large gene expression profiling study of primary human cutaneous melanomas, 254 genes with impact on metastatic dissemination were characterized [21]. In this study, immunohistochemical validation identified MCM4 and MCM6 as independent predictors of patient survival. The MCMs are subunits of the minichromosome maintenance protein complex, MCM2-7, involved in DNA replication and expressed in all phases of the cell cycle: G1, S, mitosis and G2 [22,23]. In another recent gene expression study [24], mitosin was among the genes that were up-regulated in melanoma metastases compared to primary melanomas. Mitosin, also termed centromere protein F (CENP-F), is associated with the centromere/kinethocore complex and is expressed in all active phases of the cell cycle, with a maximum in G2 and M [25]. Immunohistochemical studies on breast cancer have shown elevated expression of mitosin to be correlated with poor prognosis [26,27].

Cdc6 has previously been reported as a p16 suppressor [28]. This protein is a regulator of the cell cycle and thereby cell proliferation by inhibition of Cyclin-Cdk4/6 complex formation [29]. In our series, loss of p16 expression is previously shown to be associated with increased Ki-67 expression and poor outcome [13].

On this background, the aim of our present study was to evaluate the prognostic impact of mitotic count, Ki-67 expression and novel proliferation markers PHH3, MCM4, mitosin and Cdc6, and to compare the results with important histopathological variables.

## Methods

### Patients

The patient material of this series is described in detail elsewhere [13]. Briefly, 202 consecutive cases of vertical growth phase melanoma of the nodular type occurring in the period 1981 to 1997 were initially included (median age 64.4 years, median thickness 3.6 mm, range from 1.0 mm to 44.0 mm). The presence of a vertical growth phase and the lack of a radial growth phase, *i.e. *adjacent in situ or microinvasive components, were used as inclusion criteria. Cases with minor secondary involvement of the adjacent epidermis up to three epidermal ridges were included. There was no known history of familial occurrence. In addition, 58 paired metastases (local skin, regional lymph nodes, distant) were examined. During this period, the sentinel node procedure was not performed.

Complete information on patient survival and time and cause of death was available in all 202 cases. Last date of follow-up was December 31, 1999, and median follow-up time for survivors was 89 months (range from 24 to 221). Recurrence-free survival was available in 167 of 202 patients. During the follow-up period, 72 patients (36%) died of malignant melanoma, and 45 (22%) died of other causes. Of the 167 radically treated patients with data on recurrence-free survival, 74 (44%) had recurrent disease.

Previously reported information on clinico-pathological characteristics and survival data [13], the proliferation marker Ki-67 [13], cell cycle regulators [13,30,31] and EZH2 expression [32] were included for comparison.

In addition to this series of nodular melanoma, 32 cases of benign melanocytic nevi (median age 26.6 years) and 20 consecutive cases of invasive superficial spreading melanomas >1 mm in thickness (median age 49.0 years, median thickness 1.7 mm) occurring in the period 1981 to 1983 were included to examine various markers in different categories of melanocytic lesions.

In this study, mitotic count and Ki-67 expression [13] were assessed on standard sections of all the 202 cases of primary malignant melanoma. Tissue microarray (TMA) sections were used for examination of PHH3, MCM4, mitosin and Cdc6; 96-129 cases had sufficient material left in the TMA blocks for these markers. Comparisons between regular slides and TMA sections were done (see Results).

TMA sections of benign nevi, superficial spreading melanomas and metastases were stained for PHH3, MCM4, mitosin and Cdc6. Mitotic count was not examined in these cases. Ki-67 expression was examined in standard sections of the metastases, but not in benign nevi or superficial spreading melanomas.

The Norwegian Data Inspectorate and the Regional Committee for Ethics in Research (Health Region III) (178.05) have approved this project. The study was performed in accordance with the Helsinki Declaration.

### Clinico-pathological variables

The following variables were recorded: date of histological diagnosis, sex, age at diagnosis, anatomical site of the primary tumor, and presence of metastases at diagnosis (local, regional, distant). The H&E-stained slides were previously re-examined, and the following histological features included: tumor thickness according to Breslow [33], level of invasion according to Clark [34], microscopic tumor ulceration [35], necrosis [36], and vascular invasion [35].

#### Mitotic count

The mitotic count was recorded on H&E stained sections using light microscope (×400). Mitotic figures were counted at the base of the tumors in the most active area ("hot spots") in a minimum of 3 consecutive HPFs (HPF size 0.29 mm^2^), and the number of mitosis per mm^2 ^was calculated (IMB, LAA).

### Tissue Microarray (TMA)

The TMA technique has been described and validated in several studies [37-39]. Three tissue cylinders with a diameter of 0.6 mm [38,39] from representative tumor areas identified on H&E stained slides, generally at the suprabasal areas of the primary tumors, were punched and mounted into a recipient paraffin block using a custom-made precision instrument (Beecher Instruments, Silver Spring, MD, USA). Sections of the resulting TMA blocks (5 μm) were then made by standard technique.

### Immunohistochemistry

The immunohistochemical staining was performed on thin TMA sections (5 μm) of paraffin-embedded archival tissue. The slides were dewaxed with xylene/ethanol before microwave antigen retrieval for 10 min at 750 W and 15 min at 350 W in TE 9 buffer (pH = 9) or citrate butter (pH = 6). The staining protocols were optimized for each of the antibodies. The Ki-67 staining protocol and evaluation are previously described [13].

#### PHH3

Antigen retrieval was carried out in Tris-EDTA buffer. The slides were incubated for 1 hour at room temperature with a polyclonal rabbit Phosphohistone H3 antibody (Ser 10), catalogue # 06-570 (Millipore, Billerica, MA, USA), diluted 1:1500.

#### MCM4

The microwave treatment was performed in Tris-EDTA buffer, before incubation for 1 hour at room temperature with a monoclonal mouse MCM4 antibody, catalogue # sc-28317 (Santa Cruz Biotechnology, Santa Cruz, CA, USA), diluted 1:25.

#### Mitosin

After microwave treatment in citrate buffer, the TMA slides were incubated overnight at 4°C with the polyclonal rabbit CENPF antibody, catalogue # ab5 (Abcam, Cambridge, UK), diluted 1:100.

#### Cdc6

Microwave antigen retrieval was performed in citrate buffer, followed by incubation overnight at 4°C with the monoclonal mouse Cdc6 antibody, catalogue # sc-9964 (Santa Cruz Biotechnology, Santa Cruz, CA, USA), diluted 1: 20.

The staining procedures were performed using the EnVision labelled polymer method (Dako, Copenhagen, Denmark), with 3-amino-9-ethylcarbazole (AEC) peroxidase as substrate before brief counterstaining with Dako REAL hematoxylin.

Negative controls were incubated with mouse IgG of the same isotype and in the same dilution in the cases of monoclonal antibodies, MCM4 and Cdc6. In the PHH3 and mitosin protocols, the negative controls were obtained by omitting the primary antibody. All turned out negative.

### Evaluation of staining

#### PHH3

**Figure 1:**
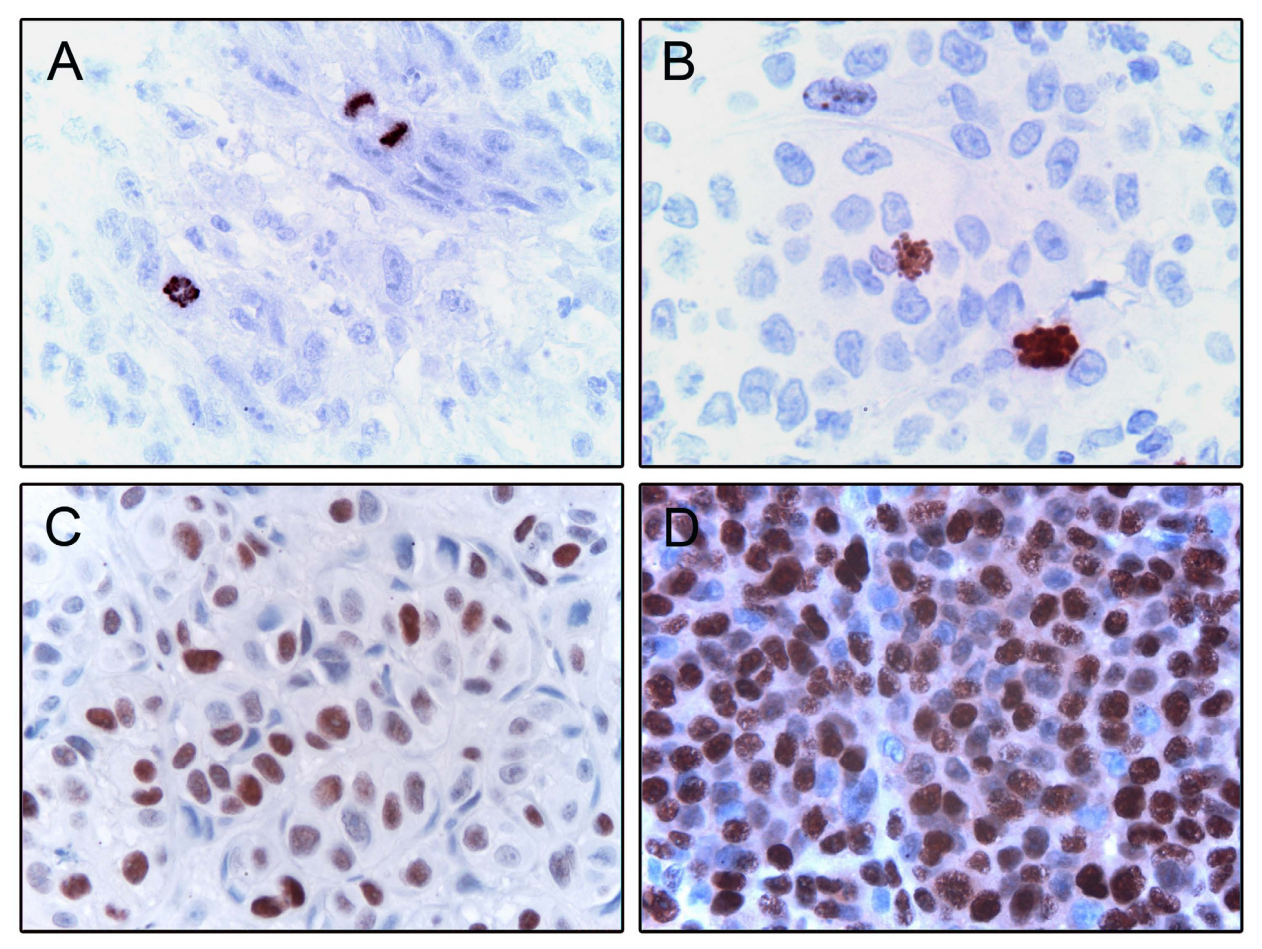
**Immunohistochemical staining showing PHH3 positive prophases and mitotic figures (A and B), high expression of MCM4 (C) and mitosin (D)**.

The staining of PHH3 was evaluated by counting of positive cells in prophase, metaphase, anaphase or telophase (Fig. 1). In general, the staining of PHH3 was strong and crisp, with no background. Some interphase nuclei showed sparse granular staining, but these were not counted. The percentage of positive tumor cell nuclei was counted in 5 × 100 cells for each case (magnification ×400, field size 0.18 mm^2^) in selected areas with most positive nuclei, *i.e. *"hot spots". A subset (n = 50) was scored blindly by two observers (RGL, IMB) showing good agreement (Spearman's correlation coefficient 0.82, p < 0.001; κ = 0.72).

The number of positive tumor cell nuclei per HPF was also estimated in all the cases, as the average of the count in 3 HPFs at ×400 magnification, and there was a significant correlation with percentage positive nuclei (Spearman's correlation coefficient 0.93, p < 0.001).

For internal validation, 25 regular slides of primary melanomas were stained for PHH3, and both the percentage of positive tumor cell nuclei and the average number of positive tumor cell nuclei per HPF correlated well with the results from the TMA sections (Spearman's correlation coefficient 0.80 and 0.74, respectively, both p < 0.001).

#### MCM4

Staining of MCM4 was mainly nuclear (Fig. 1). Evaluation was assessed by recording the percentage positive tumor cell nuclei as described for PHH3. A subset (n = 22) was examined blindly by two of the authors (RGL, IMB) (Spearman's correlation coefficient 0.94, p < 0.001; κ = 0.70).

#### Mitosin

The nuclear immunohistochemical staining of mitosin was generally fairly strong (Fig. 1), and recorded using a semi-quantitative and subjective grading, considering both the intensity of staining and the proportion of tumor cell nuclei showing unequivocal positive reaction. A staining index (SI) was calculated as a product of staining intensity (0-3) and area of positive tumor cell; 1 < 10%, 2 = 10-50%, 3 > 50%; [32]. The staining of all primary melanomas was scored blindly by two investigators (κ = 0.57). In the cases of disagreement, slides were re-evaluated by both observers (RGL, IMB) to obtain a consensus.

#### Cdc6

The staining of Cdc6 in tumor cells was primarily cytoplasmic and recorded by the same grading system as mitosin, with the difference that cytoplasmic staining intensity and area was evaluated instead of the nuclear staining. There was good correlation regarding the assessment of the staining index in primary melanomas (n = 116) by two observers (RGL, IMB) (κ = 0.74).

Evaluation of the cases was done blinded for patient characteristics and outcome. In subsequent statistical analyses, the cut-off point for categorization was based on the median values for PHH3 and MCM4, the lower quartile SI for mitosin and the median SI for Cdc6, after considering the frequency distribution curve and size of subgroups as well as number of events. PHH3 and MCM4 were categorized as low and high by the median percentage of positive tumor cell nuclei, 0.8% and 40.7%, respectively. Mitosin and Cdc6 were categorized as low and high, with cut-off points at SI = 6 and SI = 4, respectively.

### Statistics

Analyses were performed using the SPSS statistical package, version 15.0 (SPSS Inc, Chicago, IL). Associations between different categorical variables were assessed by Pearson's chi-square test. Continuous variables not following the normal distribution were compared between two or more groups using the Mann-Whitney U or Kruskal-Wallis H tests. Wilcoxon signed rank test was used to compare related samples. Nonparametric correlations were tested by the Spearman's rank coefficient, and Kappa (κ) statistics was used in analyses of inter-observer agreement of categorical data.

Univariate analyses of time to death due to malignant melanoma or time to recurrence (recurrence-free survival) were performed using the product-limit procedure (Kaplan-Meier method), and differences between categories were estimated by the log-rank test, with date of histological diagnosis as the starting point. Patients who died of other causes were censored at the date of death. The influence of co-variates on patient survival and recurrence-free survival was analysed by the proportional hazards method, and tested by the likelihood ratio (lratio) test. All results were considered significant if p ≤ 0.05.

## Results

### Primary nodular melanoma

#### Mitotic count

**Figure 2:**
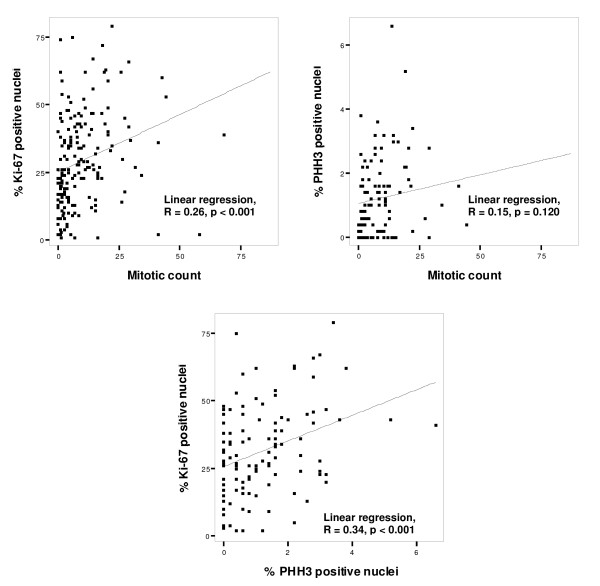
**Scatter plots with regression lines illustrating the relationships between mitotic count (mitosis/mm^2^), Ki-67 expression and PHH3 expression**.

The median value of the mitotic count was 6 mitotic figures per mm^2^. Increased mitotic count was strongly associated with tumor thickness, presence of ulceration and tumor necrosis (all p < 0.001; Kruskal-Wallis or Mann-Whitney test), as well as advanced Clark's level of invasion (p = 0.038) and presence of vascular invasion (p = 0.052) (Table 1). In addition, high mitotic count was associated with elevated Ki-67 expression in tumor cells (p < 0.001, R = 0.26 by linear regression, as illustrated in Fig. 2). Among cell cycle regulators, increased mitotic count was associated with strong EZH2 expression (p = 0.001, Mann-Whitney test; data not shown).

**Table 1:** Associations between different proliferation markers and histopathological variables

			Mitotic count			Ki-67		PHH3		MCM4		Mitosin		
			**(no./mm**^**2**^**)**			(% pos)		(% pos)		(% pos)		**(n**^**c**^**)**		
			median		**p-value**^**a**^	median	**p-value**^**a**^	median	**p-value**^**a**^	median	**p-value**^**a**^	low	high	**p-value**^**b**^
Tumor thickness					<0.001		<0.001		0.031		ns			ns
≤2.0 mm			1.7			19.0		0.4		43.0		4	7	
2.1-4.0 mm			4.3			26.5		0.8		33.2		17	23	
>4.0 mm			9.4			35.0		1.0		44.4		24	28	
											
Tumor ulceration					<0.001		<0.001		0.041		0.003			ns
absent			4.3			23.5		0.6		32.0		18	26	
present			9.4			33.0		1.4		46.4		27	30	
											
Tumor necrosis					<0.001		0.007		0.027		ns			ns
absent			4.3			25.0		0.6		37.4		26	38	
present			12.0			35.0		1.6		45.2		19	20	
											
Clark's level					0.038		0.015		ns		ns			ns
II - IV			5.1			26.0		0.8		37.5		35	45	
V			7.7			35.0		1.0		47.2		10	13	
											
Vascular invasion					0.052		0.027		ns		ns			ns
absent			5.1			26.0		0.8		40.6		35	42	
present			8.15			34.5		1.0		41.4		10	16	

^a^Mann-Whitney or Kruskal-Wallis test

^b^Chi-square test

^c^Staining index, cut-off point lower quartile

#### Ki-67

The median percentage of Ki-67 expression was 27% (range from 1 to 79%). Elevated Ki-67 expression was correlated with increased tumor thickness and presence of tumor ulceration (both p < 0.001; Kruskal-Wallis or Mann-Whitney test), tumor necrosis (p = 0.007), increased Clark's level of invasion (p = 0.015) and vascular invasion (p = 0.027) (Table 1). Furthermore, the level of Ki-67 expression was correlated with high EZH2 expression (p = 0.002, Mann-Whitney test; data not shown).

#### PHH3

The range of percentage PHH3 positive tumor nuclei was from 0.0 to 6.6% (median value 0.8%). Increased expression of PHH3 was significantly associated with tumor thickness (p = 0.031), presence of tumor ulceration (p = 0.041) and tumor necrosis (p = 0.027), but not with Clark's level of invasion (Table 1). High levels of PHH3 was associated with increased mitotic count (p = 0.003) and high Ki-67 expression (p = 0.002) (Table 2). In addition, elevated PHH3 was associated with high levels of EZH2 expression (p < 0.001, Mann-Whitney test; data not shown).

**Table 2:** Mitotic count and Ki-67 in association with expression of PHH3, MCM4 and mitosin

	Mitotic count		Ki-67	
	**(no./mm**^**2**^**)**		(% pos)	
	median	**p-value**^**a**^	median	**p-value**^**a**^
PHH3 (% pos)		0.003		0.002
≤0.8^b^	3.4		26.0	
>0.8	8.9		36.0	
				
MCM4 (% pos)		0.002		0.001
≤40.7^b^	5.1		26.0	
>40.7	11.1		37.5	
				
Mitosin (SI^d^)		ns		ns
low^c^	9.4		34.5	
high	7.7		31.5	

^a^Mann-Whitney test

^b^Cut-off point median

^c^Cut-off point lower quartile

^d^Abbreviation: SI, staining index

Using regular slides, the recalculated median value of positive PHH3 nuclei per mm^2 ^was 20.9; the median value of the mitotic count was 8.6 per mm^2 ^in the same subset (n = 25) of the primary melanomas. Thus, PHH3 count was 2.4 times higher than the mitotic count.

#### MCM4

The proportion of positively stained MCM4 in tumor cell nuclei was ranging from 0.0 to 84.8% (median value 40.7%). High expression of MCM4 was correlated with tumor ulceration (p = 0.003), high mitotic count (p = 0.002), but not with other histopathological variables (Table 1, 2). Elevated MCM4 expression was also associated with elevated Ki-67 expression (p = 0.001) (Table 2). Increased MCM4 expression and elevated PHH3 expression was correlated (Spearman's correlation coefficient 0.55, p < 0.001). Further, high MCM4 expression was correlated to high EZH2 expression (p < 0.001, Mann-Whitney test; data not shown).

#### Mitosin

Mitosin was not correlated with mitotic count, expression of Ki-67, cell cycle regulators, nor any of the investigated histopathological variables.

#### Cdc6

Cdc6 was not correlated with mitotic count, Ki-67 expression, MCM4 or mitosin. In the group with high Cdc6 expression, there was significant higher level of PHH3 positivity than the group with low Cdc6 expression (p = 0.042, Mann-Whitney test; data not shown). Notably, the level of Cdc6 was not correlated with p16 expression in our series. There was no association between level of Cdc6 expression and histopathological variables.

### Primary and metastatic melanoma

The Ki-67 expression in metastases was significantly higher than in the corresponding primary melanomas, with a median value of 43% positivity in metastases compared with 27% in primary nodular melanomas (p = 0.001, Wilcoxon signed ranks test).

There was no significant difference in PHH3 and Cdc6 levels in metastases compared to corresponding primary tumors, whereas MCM4 and mitosin were significantly lower in metastases (p < 0.001 and p = 0.012, respectively; Wilcoxon signed ranks test).

### Melanocytic nevi and melanomas

PHH3 expression was higher in melanomas (nodular and superficial combined, n = 127), when compared with benign nevi (n = 32) (p < 0.001, Mann-Whitney test). The median values of percentage positive PHH3 nuclei in the two groups were 0.8 and 0.0, respectively.

In the melanoma group (n = 113), the median value of MCM4 positivity was 36.8% in contrast to 2.0% in nevi (n = 31) (p < 0.001, Mann-Whitney test).

Mitosin and Cdc6 were also significantly higher in the group of nodular and superficial melanomas than in nevi (p < 0.001, Pearson's chi square test).

### Survival analyses

**Figure 3:**
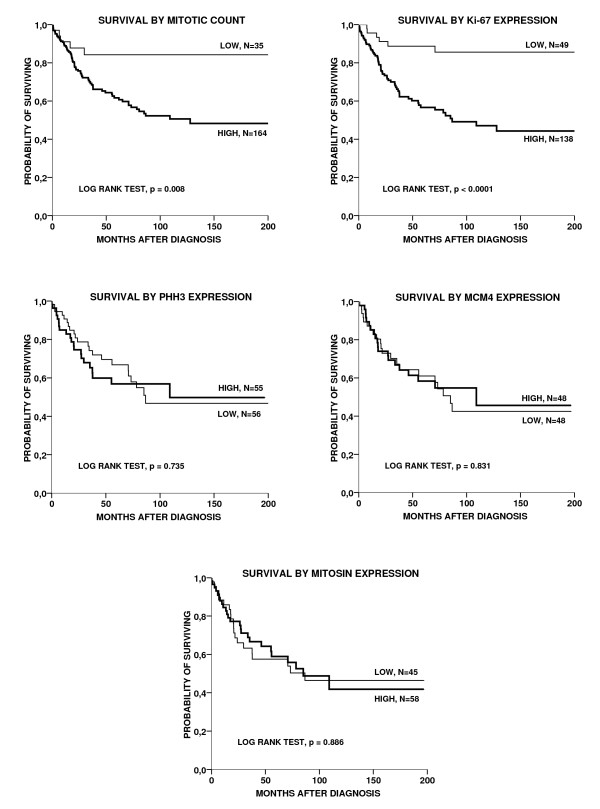
**Survival curves according to the Kaplan-Meier method with cut-off points at the lower quartile for mitotic count (<1.7 mitotic figures/mm^2^), Ki-67 expression (≤16% positive tumor cell nuclei) and mitosin (SI ≤ 6), and at the median values for PHH3 (≤0.8% positive nuclei) and MCM4 (≤40.7% positive nuclei)**.

Univariate survival analysis showed worse outcome in the group of patients with high mitotic count (p = 0.008), using a cut-off at the lower quartile (mitotic count ≥ 1.7 per mm^2^) (Fig. 3; Table 3). In the subgroup of cases with high mitotic count, 5-year survival was 62% and 10-year survival was 51%. In contrast, both 5-year and 10-year survival were 84% among the rest. Further, univariate survival analysis showed significant influence of Ki-67 expression (p < 0.0001) (Fig. 3; Table 3). In the cases with high Ki-67 expression (>16% by lower quartile), 5-year survival was 57% and the 10-year survival was 47%. In the group with low Ki-67 positivity, the 5- and 10 year survival were 89% and 86%, respectively. In contrast, the survival analyses did not show any significant influence of PHH3, MCM4, mitosin or Cdc6 on outcome (Fig. 3; Table 3).

**Table 3:** Univariate survival analysis according to histopathological variables and proliferation markers

Variables	n	Estimated survival rates (%)		**p-value**^**a**^
			
		5 years	10 years	
Tumor thickness				0.001
≤2.0 mm	48	80	77	
2.1-4.0 mm	73	76	59	
>4.0 mm	81	49	43	
				
Tumor ulceration				0.001
absent	114	77	68	
present	83	54	44	
				
Clark's level				<0.001
II - IV	166	72	62	
V	35	40	32	
				
Vascular infiltration				<0.001
absent	162	73	61	
present	40	39	39	
				
Tumor necrosis				<0.001
absent	142	76	64	
present	57	39	36	
				
Mitotic count (no./mm^2^)				0.008
<1.7^b^	35	84	84	
≥1.7	164	62	51	
				
Ki-67 (% pos)				<0.0001
≤16^b^	49	89	86	
>16	138	57	47	
				
PHH3 (% pos)				ns
≤0.8^c^	56	67	47	
>0.8	55	57	50	
				
MCM4 (% pos)				ns
≤40.7^c^	48	61	43	
>40.7	48	58	46	
				
Mitosin (SI^d^)				ns
low^b^	45	58	47	
high	58	59	42	

^a^Log-rank test

^b^Cut-off point lower quartile

^c^Cut-off point median

^d^Abbreviation: SI, staining index

In the multivariate analysis (proportional hazards method), we first examined the histopathological variables included in the pT system from 2010, tumor thickness, tumor ulceration, and mitotic count. In this basic model, tumor thickness >4.0 mm was a predictor of poor prognosis compared with tumors ≤2.0 mm (HR = 2.4, p = 0.020). Presence of ulceration (HR = 1.7; p = 0.028) was also a prognostic indicator in this model, whereas mitotic count was not, even if it was included as a continuous variable, or if other cut-points were used. Next, the same variables were included together with Ki-67 expression in a new model (Table 4). Tumor thickness >4.0 mm compared with ≤2.0 mm (p = 0.041) and level of Ki-67 expression (p = 0.003) were significant predictors of prognosis in this model, whereas ulceration and mitotic count were not. Further, we also included other histopathological variables that were previously shown to be strong prognostic indicators in this series by univariate analysis, i.e. Clark's level of invasion, vascular infiltration and tumor necrosis (Table 5). In this model, Clark's level (p = 0.009), vascular invasion (p = 0.008), tumor necrosis (p = 0.015), and Ki-67 expression (p = 0.006) showed prognostic impact, whereas tumor thickness, ulceration, and mitotic count did not. Exclusion of Ki-67 did not influence the impact of mitotic count in the model.

**Table 4:** Multivariate survival analysis (Cox' proportional hazards method)

Variable	n	**HR**^**a**^	95% CI		**p-value**^**b**^
Tumor thickness					0.073
≤2.0 mm	43	1			
2.1-4.0 mm	65	1.7	0.7 to 4.1		ns
>4.0 mm	72	2.5	1.04 to 5.8		0.041
				
Tumor ulceration					0.083
absent	102	1			
present	78	1.6	0.9 to 2.6		
				
Mitotic count (no./mm^2^)					ns
<1.7^c^	34	1			
≥1.7	146	1.5	0.6 to 3.9		
				
Ki-67 (% pos)					0.003
≤16^c^	48	1			
>16	132	3.1	1.3 to 7.4		

^a^Hazard ratio

^b^Likelihood ratio or Wald test

^c^Cut-off point lower quartile

**Table 5:** Multivariate survival analysis (Cox' proportional hazards method)

Variable	n	**HR**^**a**^	95% CI		**p-value**^**b**^
Tumor thickness					ns
≤2.0 mm	43	1			
2.1-4.0 mm	64	1.3	0.5 to 3.2		ns
>4.0 mm	72	1.1	0.4 to 2.9		ns
				
Tumor ulceration					ns
absent	101	1			
present	78	1.3	0.7 to 2.3		
				
Clark's level					0.009
II - IV	146	1			
V	33	2.5	1.3 to 4.9		
				
Vascular infiltration					0.008
absent	143	1			
present	36	2.3	1.3 to 4.0		
				
Tumor necrosis					0.015
absent	127	1			
present	52	2.1	1.2 to 3.8		
				
Mitotic count (no./mm^2^)					ns
<1.7^c^	34	1			
≥1.7	145	1.5	0.5 to 3.9		
				
Ki-67 (% pos)					0.006
≤16^c^	48	1			
>16	131	3.0	1.3 to 7.3		

^a^Hazard ratio

^b^Likelihood ratio or Wald test

^c^Cut-off point lower quartile

If tumor thickness was included as a continuous variable, thickness was a significant prognostic indicator, also in the model shown in Table 5. Additional analyses showed that this was due to a small subgroup (n = 13) of tumors >10 mm with very poor prognosis. However, the prognostic impact of mitotic count and Ki-67 were not altered if thickness was used as a continuous variable.

In this series of nodular melanomas, location on the trunk compared with other locations had significant prognostic impact in univariate (p < 0.001) and multivariate analyses (HR = 2.6, p = 0.002, if added to the model shown in Table 5), but it did not influence the impact of mitotic count or Ki-67 expression. Age and sex were not correlated with survival in univariate analyses, and did not alter the multivariate survival analyses if included.

## Discussion

Increased tumor cell proliferation is a hallmark of malignancy [40] and also a prognostic factor in multiple tumors, among these cutaneous melanoma. Assessment of proliferation may facilitate more accurate staging of melanomas, and thereby be an aid in the selection of patients for treatment.

In the present study of thick cutaneous melanomas, increased mitotic count and Ki-67 expression were both associated with known unfavourable features, such as increased tumor thickness, Clark's levels of invasion, presence of tumor ulceration, tumor necrosis and vascular invasion. High mitotic count was a predictor of poor prognosis in univariate analysis, while increased percentage of Ki-67 positivity in tumor cells was stronger in multivariate survival models.

Mitotic count has shown an independent prognostic value in several studies of primary melanoma [6,7,10], whereas others have shown prognostic significance of mitotic count in univariate analysis only [8,41], as in the present study. Some authors claim that mitotic count should not be incorporated in the routine staging system due to limited additional prognostic information [41]. The reproducebility and the time consume are also important aspects to consider [41]. Nevertheless, the majority of authors seem to be of the opinion that assessment of mitoses should be incorporated in the staging of cutaneous melanoma [6-11]. Therefore, in the latest AJCC classification system from 2010, mitotic count has been included in the substaging of pT1 tumors.

Ki-67 has been the most widely used proliferation marker in melanomas and other tumors. In a recent review, Ki-67 staining was considered a helpful supplement in distinguishing benign from malignant melanocytic lesions, but the prognostic value is more uncertain in melanomas [12]. Still, in a systematic review and meta-analysis of published literature on prognostic immunohistochemical biomarkers in cutaneous melanoma, Ki-67 was among the most promising [42]. This analysis included the previously published data from the present study [13]. A limitation in the use of Ki-67 is the lack of methodological standardization and a validated cut-off level. This is a possible bias in comparing different studies, and restricts the utility in a routine setting. Thus, alternative methods of proliferation assessment are of interest, in particular if they could be used in routine practice. Since only a subset of the cells expressing Ki-67 in fact will go through mitosis [43], one important aim of this study was to investigate whether mitotic count was a better way to estimate proliferation and prognosis, and to compare the impact of Ki-67 with mitotic count and a selection of more recently introduced proliferation markers.

To the best of our knowledge, there are few previous studies on cutaneous melanomas with data on both mitotic count and Ki-67 expression included in multivariate prognostic analyses, and no such studies on thick melanomas. A cohort study by Gimotty et al. on 396 thin melanomas (tumor thickness ≤1 mm) with at least 10 years follow-up, showed mitotic count ≥1 per mm^2 ^and Ki-67 expression in ≥20% of tumor cell nuclei to be independent prognostic factors of metastases [9]. Presence of mitoses had the strongest prognostic impact. In another study of 169 thin melanomas, Frahm et al. found 5 cases with high Ki-67 expression (≥25% positive tumor cells) of which 4 developed metastases, and in the multivariate analyses, no other factors, included mitotic count, turned out to be independently predictive [44].

Mitosis can be difficult to distinguish from apoptotic figures. In this respect, the mitotic marker anti-PHH3 is interesting, as it lightens up mitosis that appear as sharply stained figures, whereas apoptotic cells remain negative [19,45]. Especially, prophases are emphasized, in line with previous reports [45]. In the present study, PHH3 was associated with tumor thickness, ulceration and necrosis in the group of nodular melanomas. Since PHH3 correlated significantly with mitotic count and Ki-67 expression, it was surprising that the PHH3 frequency did not show an association with patient outcome. An obvious objection and possible weakness in the method was that mitotic count and Ki-67 was assessed on whole sections, while PHH3 staining was performed on TMA sections. However, there was a good correlation between the PHH3 frequency in TMA and standard sections.

The median number of PHH3 positive cells per mm^2 ^was higher than the median value of the mitotic count per mm^2 ^in the regular slides (ratio 2.4). This is expected, as fewer mitotic figures should be missed when they are highlighted by staining, and prophases are impossible to count on standard HE-sections. Corresponding results with increased number of identified mitosis by using PHH3 staining are described in other tumor types such as breast cancer [17,19,45,46]. Based on these findings, PHH3 frequency does not appear to be a useful proliferation marker in cutaneous melanoma, although we believe that this should be studied in more detail.

MCM4 was associated with mitotic count and Ki-67 expression, but there was no influence on survival related to the level of MCM4 expression. In the metastases, MCM4 staining was actually lower than in primary tumors. Thus, these findings did not validate MCM4 as a prognostic marker in cutaneous melanoma as reported by Winnepenninckx [21].

Mitosin did not turn out as a useful proliferation marker in the present study, as it was not correlated to any of the investigated variables, including Ki-67 and mitotic count, nor to patient survival. Further, our results did not confirm an increased expression in metastases as compared with primary melanomas as described in a gene expression study of non-paired primary tumors and metastases [24]. In contrast, we found that expression of mitosin was lower in the metastases. In a study of breast cancer which showed a prognostic value of mitosin [26], the same CENPF antibody in equivalent dilution was used.

The present study did not show an inverse association between expression of Cdc6 and the p16 tumor suppressor protein. Further, Cdc6 was not a useful proliferation marker, despite being correlated with PHH3, with no prediction of survival. Jaeger et al. identified Cdc6 among upregulated genes in melanoma metastases compared to primary tumors as validated by immunohistochemistry [24]. Cdc6 was also in the group of genes associated with an unfavourable prognosis in a different gene expression study by Winnepenninckx et al [21]. However, in a subsequent immunohistochemical staining on TMA, Cdc6 was not a significant prognostic predictor, in line with our findings.

Strong EZH2 expression was associated with increased Ki-67 staining and mitotic counts as previously reported [32]. Also, high levels of PHH3 and elevated MCM4 expression were significantly correlated with strong EZH2. Association between proliferation by Ki-67 and expression of EZH2 has been shown in several other tumors such as prostate [32], breast [47], salivary gland adenoid cystic carcinoma [48], and oral squamous cell carcinoma [49]. EZH2 promotes S-phase entry and G2-M transition [50,51], and the epigenetic modifying effects of EZH2 histone methyltransferase is involved in silencing of cell cycle control genes [52].

## Conclusions

In conclusion, Ki-67 was a stronger and more robust prognostic indicator than mitotic count in this series of nodular melanomas (median thickness 3.6 mm). Although the novel proliferation markers PHH3 and MCM4 were significantly correlated with Ki-67 and mitotic counts, they did not predict patient survival. Therefore, Ki-67 expression was a superior proliferation marker in this series of thick cutaneous melanoma

## Competing interests

The authors declare that they have no competing interests.

## Authors' contributions

LAA contributed to design of the study, histological review of the cases, interpretation of data, writing of the manuscript, and provided the funding. IMB participated in study design, laboratory work, interpretation of data, statistical analyses, and writing of the manuscript. OS made the tissue arrays, collected follow-up data and patients demographics, and participated in laboratory work. RGL contributed to study design, laboratory work, interpretation of data, statistical analyses, and writing of the manuscript. All authors read and approved the final manuscript.

## Pre-publication history

The pre-publication history for this paper can be accessed here:

http://www.biomedcentral.com/1471-2407/10/140/prepub

## References

[B1] Beddingfield FC III The melanoma epidemic: res ipsa loquitur Oncologist 2003 8 5 459 465 10.1634/theoncologist.8-5-459 14530499

[B2] Thompson JF Scolyer RA Kefford RF Cutaneous melanoma Lancet 2005 365 9460 687 701 15721476 10.1016/S0140-6736(05)17951-3

[B3] Tsao H Atkins MB Sober AJ Management of cutaneous melanoma N Engl J Med 2004 351 10 998 1012 10.1056/NEJMra041245 15342808

[B4] Balch CM Soong SJ Gershenwald JE Thompson JF Reintgen DS Cascinelli N Urist M McMasters KM Ross MI Kirkwood JM Prognostic factors analysis of 17,600 melanoma patients: validation of the American Joint Committee on Cancer melanoma staging system J Clin Oncol 2001 19 16 3622 3634 11504744 10.1200/JCO.2001.19.16.3622

[B5] Balch CM Buzaid AC Soong SJ Atkins MB Cascinelli N Coit DG Fleming ID Gershenwald JE Houghton A Jr Kirkwood JM Final version of the American Joint Committee on Cancer staging system for cutaneous melanoma J Clin Oncol 2001 19 16 3635 3648 11504745 10.1200/JCO.2001.19.16.3635

[B6] Barnhill RL Katzen J Spatz A Fine J Berwick M The importance of mitotic rate as a prognostic factor for localized cutaneous melanoma J Cutan Pathol 2005 32 4 268 273 10.1111/j.0303-6987.2005.00310.x 15769275

[B7] Francken AB Shaw HM Thompson JF Soong SJ Accortt NA Azzola MF Scolyer RA Milton GW McCarthy WH Colman MH The prognostic importance of tumor mitotic rate confirmed in 1317 patients with primary cutaneous melanoma and long follow-up Ann Surg Oncol 2004 11 4 426 433 10.1245/ASO.2004.07.014 15070604

[B8] Nagore E Oliver V Botella-Estrada R Moreno-Picot S Insa A Fortea JM Prognostic factors in localized invasive cutaneous melanoma: high value of mitotic rate, vascular invasion and microscopic satellitosis Melanoma Res 2005 15 3 169 177 10.1097/00008390-200506000-00005 15917698

[B9] Gimotty PA Van Belle P Elder DE Murry T Montone KT Xu X Hotz S Raines S Ming ME Wahl P Biologic and prognostic significance of dermal Ki67 expression, mitoses, and tumorigenicity in thin invasive cutaneous melanoma J Clin Oncol 2005 23 31 8048 8056 10.1200/JCO.2005.02.0735 16258103

[B10] Azzola MF Shaw HM Thompson JF Soong SJ Scolyer RA Watson GF Colman MH Zhang Y Tumor mitotic rate is a more powerful prognostic indicator than ulceration in patients with primary cutaneous melanoma: an analysis of 3661 patients from a single center Cancer 2003 97 6 1488 1498 10.1002/cncr.11196 12627514

[B11] Ross MI Early-stage melanoma: staging criteria and prognostic modeling Clin Cancer Res 2006 12 7 Pt 2 2312s 2319s 10.1158/1078-0432.CCR-05-2643 16609051

[B12] Ohsie SJ Sarantopoulos GP Cochran AJ Binder SW Immunohistochemical characteristics of melanoma J Cutan Pathol 2008 35 5 433 444 10.1111/j.1600-0560.2007.00891.x 18399807

[B13] Straume O Sviland L Akslen LA Loss of nuclear p16 protein expression correlates with increased tumor cell proliferation (Ki-67) and poor prognosis in patients with vertical growth phase melanoma Clin Cancer Res 2000 6 5 1845 1853 10815907

[B14] Hendzel MJ Wei Y Mancini MA Van Hooser A Ranalli T Brinkley BR Bazett-Jones DP Allis CD Mitosis-specific phosphorylation of histone H3 initiates primarily within pericentromeric heterochromatin during G2 and spreads in an ordered fashion coincident with mitotic chromosome condensation Chromosoma 1997 106 6 348 360 10.1007/s004120050256 9362543

[B15] Juan G Traganos F James WM Ray JM Roberge M Sauve DM Anderson H Darzynkiewicz Z Histone H3 phosphorylation and expression of cyclins A and B1 measured in individual cells during their progression through G2 and mitosis Cytometry 1998 32 2 71 77 10.1002/(SICI)1097-0320(19980601)32:2<71::AID-CYTO1>3.0.CO;2-H 9627219

[B16] Hendzel MJ Nishioka WK Raymond Y Allis CD Bazett-Jones DP Th'ng JP Chromatin condensation is not associated with apoptosis J Biol Chem 1998 273 38 24470 24478 10.1074/jbc.273.38.24470 9733739

[B17] Kim YJ Ketter R Steudel WI Feiden W Prognostic significance of the mitotic index using the mitosis marker anti-phosphohistone H3 in meningiomas Am J Clin Pathol 2007 128 1 118 125 10.1309/HXUNAG34B3CEFDU8 17580279

[B18] Takei H Bhattacharjee MB Rivera A Dancer Y Powell SZ New immunohistochemical markers in the evaluation of central nervous system tumors: a review of 7 selected adult and pediatric brain tumors Arch Pathol Lab Med 2007 131 2 234 241 17284108 10.5858/2007-131-234-NIMITE

[B19] Skaland I Janssen EA Gudlaugsson E Klos J Kjellevold KH Soiland H Baak JP Phosphohistone H3 expression has much stronger prognostic value than classical prognosticators in invasive lymph node-negative breast cancer patients less than 55 years of age Mod Pathol 2007 20 12 1307 1315 10.1038/modpathol.3800972 17917671

[B20] Nasr MR El-Zammar O Comparison of pHH3, Ki-67, and survivin immunoreactivity in benign and malignant melanocytic lesions Am J Dermatopathol 2008 30 2 117 122 10.1097/DAD.0b013e3181624054 18360113

[B21] Winnepenninckx V Lazar V Michiels S Dessen P Stas M Alonso SR Avril MF Ortiz Romero PL Robert T Balacescu O Gene expression profiling of primary cutaneous melanoma and clinical outcome Journal of the National Cancer Institute 2006 98 7 472 482 16595783 10.1093/jnci/djj103

[B22] Bell SP Dutta A DNA replication in eukaryotic cells Annu Rev Biochem 2002 71 333 374 10.1146/annurev.biochem.71.110601.135425 12045100

[B23] Tachibana KE Gonzalez MA Coleman N Cell-cycle-dependent regulation of DNA replication and its relevance to cancer pathology J Pathol 2005 205 2 123 129 10.1002/path.1708 15643673

[B24] Jaeger J Koczan D Thiesen HJ Ibrahim SM Gross G Spang R Kunz M Gene expression signatures for tumor progression, tumor subtype, and tumor thickness in laser-microdissected melanoma tissues Clin Cancer Res 2007 13 3 806 815 10.1158/1078-0432.CCR-06-1820 17289871

[B25] Zhu X Mancini MA Chang KH Liu CY Chen CF Shan B Jones D Yang-Feng TL Lee WH Characterization of a novel 350-kilodalton nuclear phosphoprotein that is specifically involved in mitotic-phase progression Mol Cell Biol 1995 15 9 5017 5029 7651420 10.1128/mcb.15.9.5017 PMC230749

[B26] O'Brien SL Fagan A Fox EJ Millikan RC Culhane AC Brennan DJ McCann AH Hegarty S Moyna S Duffy MJ CENP-F expression is associated with poor prognosis and chromosomal instability in patients with primary breast cancer Int J Cancer 2007 120 7 1434 1443 10.1002/ijc.22413 17205517 PMC4972098

[B27] Clark GM Allred DC Hilsenbeck SG Chamness GC Osborne CK Jones D Lee WH Mitosin (a new proliferation marker) correlates with clinical outcome in node-negative breast cancer Cancer Res 1997 57 24 5505 5508 9407959

[B28] Gonzalez S Klatt P Delgado S Conde E Lopez-Rios F Sanchez-Cespedes M Mendez J Antequera F Serrano M Oncogenic activity of Cdc6 through repression of the INK4/ARF locus Nature 2006 440 7084 702 706 10.1038/nature04585 16572177

[B29] Foulkes WD Flanders TY Pollock PM Hayward NK The CDKN2A (p16) gene and human cancer Mol Med 1997 3 1 5 20 9132280 PMC2230107

[B30] Straume O Akslen LA Alterations and prognostic significance of p16 and p53 protein expression in subgroups of cutaneous melanoma Int J Cancer 1997 74 5 535 539 10.1002/(SICI)1097-0215(19971021)74:5<535::AID-IJC10>3.0.CO;2-5 9355977

[B31] Bachmann IM Straume O Akslen LA Altered expression of cell cycle regulators Cyclin D1, p14, p16, CDK4 and Rb in nodular melanomas Int J Oncol 2004 25 6 1559 1565 15547691

[B32] Bachmann IM Halvorsen OJ Collett K Stefansson IM Straume O Haukaas SA Salvesen HB Otte AP Akslen LA EZH2 expression is associated with high proliferation rate and aggressive tumor subgroups in cutaneous melanoma and cancers of the endometrium, prostate, and breast J Clin Oncol 2006 24 2 268 273 10.1200/JCO.2005.01.5180 16330673

[B33] Breslow A Thickness, cross-sectional areas and depth of invasion in the prognosis of cutaneous melanoma Ann Surg 1970 172 5 902 908 10.1097/00000658-197011000-00017 5477666 PMC1397358

[B34] Clark WH Jr From L Bernardino EA Mihm MC The histogenesis and biologic behavior of primary human malignant melanomas of the skin Cancer Res 1969 29 3 705 727 5773814

[B35] Straume O Akslen LA Expresson of vascular endothelial growth factor, its receptors (FLT-1, KDR) and TSP-1 related to microvessel density and patient outcome in vertical growth phase melanomas Am J Pathol 2001 159 1 223 235 11438469 10.1016/S0002-9440(10)61688-4 PMC1850434

[B36] Bachmann IM Ladstein RG Straume O Naumov GN Akslen LA Tumor necrosis is associated with increased alphavbeta3 integrin expression and poor prognosis in nodular cutaneous melanomas BMC Cancer 2008 8 362 10.1186/1471-2407-8-362 19061491 PMC2631589

[B37] Kononen J Bubendorf L Kallioniemi A Barlund M Schraml P Leighton S Torhorst J Mihatsch MJ Sauter G Kallioniemi OP Tissue microarrays for high-throughput molecular profiling of tumor specimens Nat Med 1998 4 7 844 847 10.1038/nm0798-844 9662379

[B38] Nocito A Bubendorf L Maria Tinner E Suess K Wagner U Forster T Kononen J Fijan A Bruderer J Schmid U Microarrays of bladder cancer tissue are highly representative of proliferation index and histological grade J Pathol 2001 194 3 349 357 10.1002/1096-9896(200107)194:3<349::AID-PATH887>3.0.CO;2-D 11439368

[B39] Straume O Akslen LA Importance of vascular phenotype by basic fibroblast growth factor, and influence of the angiogenic factors basic fibroblast growth factor/fibroblast growth factor receptor-1 and ephrin-A1/EphA2 on melanoma progression Am J Pathol 2002 160 3 1009 1019 11891198 10.1016/S0002-9440(10)64922-X PMC1867162

[B40] Hanahan D Weinberg RA The hallmarks of cancer Cell 2000 100 1 57 70 10.1016/S0092-8674(00)81683-9 10647931

[B41] Attis MG Vollmer RT Mitotic rate in melanoma: a reexamination Am J Clin Pathol 2007 127 3 380 384 10.1309/LB7RTC61B7LC6HJ6 17276944

[B42] Gould Rothberg BE Bracken MB Rimm DL Tissue biomarkers for prognosis in cutaneous melanoma: a systematic review and meta-analysis J Natl Cancer Inst 2009 101 7 452 474 10.1093/jnci/djp038 19318635 PMC2720709

[B43] Scholzen T Gerdes J The Ki-67 protein: from the known and the unknown J Cell Physiol 2000 182 3 311 322 10.1002/(SICI)1097-4652(200003)182:3<311::AID-JCP1>3.0.CO;2-9 10653597

[B44] Frahm SO Schubert C Parwaresch R Rudolph P High proliferative activity may predict early metastasis of thin melanomas Hum Pathol 2001 32 12 1376 1381 10.1053/hupa.2001.29658 11774172

[B45] Bossard C Jarry A Colombeix C Bach-Ngohou K Moreau A Loussouarn D Mosnier JF Laboisse CL Phosphohistone H3 labelling for histoprognostic grading of breast adenocarcinomas and computer-assisted determination of mitotic index J Clin Pathol 2006 59 7 706 710 10.1136/jcp.2005.030452 16461563 PMC1860410

[B46] Tapia C Kutzner H Mentzel T Savic S Baumhoer D Glatz K Two mitosis-specific antibodies, MPM-2 and phospho-histone H3 (Ser28), allow rapid and precise determination of mitotic activity Am J Surg Pathol 2006 30 1 83 89 10.1097/01.pas.0000183572.94140.43 16330946

[B47] Collett K Eide GE Arnes J Stefansson IM Eide J Braaten A Aas T Otte AP Akslen LA Expression of enhancer of zeste homologue 2 is significantly associated with increased tumor cell proliferation and is a marker of aggressive breast cancer Clin Cancer Res 2006 12 4 1168 1174 10.1158/1078-0432.CCR-05-1533 16489070

[B48] Vekony H Raaphorst FM Otte AP van Lohuizen M Leemans CR Waal I van der Bloemena E High expression of Polycomb group protein EZH2 predicts poor survival in salivary gland adenoid cystic carcinoma J Clin Pathol 2008 61 6 744 749 10.1136/jcp.2007.054262 18326020

[B49] Kidani K Osaki M Tamura T Yamaga K Shomori K Ryoke K Ito H High expression of EZH2 is associated with tumor proliferation and prognosis in human oral squamous cell carcinomas Oral Oncol 2009 45 1 39 46 10.1016/j.oraloncology.2008.03.016 18619895

[B50] Bracken AP Pasini D Capra M Prosperini E Colli E Helin K EZH2 is downstream of the pRB-E2F pathway, essential for proliferation and amplified in cancer EMBO J 2003 22 20 5323 5335 10.1093/emboj/cdg542 14532106 PMC213796

[B51] Varambally S Dhanasekaran SM Zhou M Barrette TR Kumar-Sinha C Sanda MG Ghosh D Pienta KJ Sewalt RG Otte AP The polycomb group protein EZH2 is involved in progression of prostate cancer Nature 2002 419 6907 624 629 10.1038/nature01075 12374981

[B52] Simon JA Lange CA Roles of the EZH2 histone methyltransferase in cancer epigenetics Mutat Res 2008 647 1-2 21 29 18723033 10.1016/j.mrfmmm.2008.07.010

